# The association between vegetarian diet and varicose veins might be more prominent in men than in women

**DOI:** 10.3389/fnut.2023.1046158

**Published:** 2023-06-01

**Authors:** Cheng-Ken Tsai, Oswald Ndi Nfor, Disline Manli Tantoh, Wen-Yu Lu, Yung-Po Liaw

**Affiliations:** ^1^Department of Public Health and Institute of Public Health, Chung Shan Medical University, Taichung, Taiwan; ^2^Division of Cardiovascular Surgery, Department of Surgery, E-Da Cancer Hospital, I-Shou University, Kaohsiung, Taiwan; ^3^Division of Cardiovascular Surgery, Department of Surgery, Zuoying Branch of Kaohsiung Armed Forces General Hospital, Kaohsiung, Taiwan; ^4^Department of Medical Imaging, Chung Shan Medical University Hospital, Taichung, Taiwan; ^5^Institute of Medicine, Chung Shan Medical University, Taichung, Taiwan

**Keywords:** vegetarian diet, varicose veins, sex, interaction, Taiwan biobank

## Abstract

**Background:**

Varicose veins (VVs), a common vascular disease is associated with a huge medical burden. The prevalence in women surpasses that in men. The role of vegetarian diets in the pathogenesis of the disease remains inconclusive. In this study, we examined the risk of VVs in vegetarian and non-vegetarian men and women.

**Methods:**

The study involved 9905 adults whose data were obtained from Taiwan Biobank between 2008 and 2020. Information on VVs, sex, and vegetarian diets was obtained from participants’ self-responses to the Taiwan Biobank questionnaires.

**Results:**

The study subjects consisted of 4,142 men and 5,763 women. About 12% of men and 35% of women had VVs. Study participants were predominantly non-vegetarians (91.84% were men and 88.24% were women). Women had a higher risk of VVs than men. The odds ratio (OR); 95% confidence interval (CI) was 3.414; 2.995–3.891. There was a significant interaction between sex and vegetarian diets (*p* = 0.0034). Women were at higher risk of VVs than men both in the vegetarian (OR = 1.877, 95% CI = 1.270–2.774) and non-vegetarian (OR = 3.674, 95% CI = 3.197–4.223) groups. Based on vegetarian diets, only vegetarian men had a higher risk of VVs (OR = 1.453, 95% CI = 1.069 to 1.976). Based on the sex-stratified model, the risk of VVs was significantly higher in vegetarian men (OR = 1.457, 95% CI = 1.072–1.979), and in vegetarian and non-vegetarian women with corresponding ORs (95% CI) of 3.101 (2.528–3.803) and 3.599 (3.140–4.124), respectively.

**Conclusion:**

Women were more susceptible to varicose veins compared to men, regardless of diet. However, in terms of diet, only men who followed a vegetarian diet were at greater risk for developing VVs.

## Introduction

Varicose veins is a common vascular disease in developed countries. (1) The disease is characterized by clearly visible, dilated, tortuous, and possibly prominent subcutaneous veins in the lower extremities. (2) Previous studies using self-reported questionnaires have estimated the prevalence of VVs at 31 and 32%, respectively. (3, 4) Chronic venous disease, specifically chronic venous insufficiency, is a significant and escalating issue in Western societies that presents challenges to both individuals and healthcare systems due to the substantial costs of treatment and the burden it places on medical resources. (5, 6) VVs is a degenerative disease that tends to worsen over time. A longitudinal study conducted in the UK revealed that approximately 50% of patients experienced a deterioration in the progression of chronic venous diseases over a 13-year period, and one-third of patients with preexisting varicose veins continued to experience exacerbated symptoms such as calf skin pigmentation or ulceration. (7) As such, in the clinical management of VVs, identifying and addressing risk factors for early intervention is a crucial measure that has the potential to reduce associated medical expenses and enhance the quality of life.

VVs can be attributed to several risk factors such as age, obesity, parity, heavy lifting, and long-standing hours. (8, 9) The consumption of a vegetarian diet has been associated with a 40% reduction in cardiovascular disease (CVD) mortality and coronary heart disease (CHD) risk. (10) Moreover, it is beneficial for preventing and treating heart failure and cerebrovascular disease. (11) In addition, a plant-based diet can lower blood pressure and lipid levels, improve weight management, and reduce the risk of metabolic syndrome and type 2 diabetes. (10)

The vegetarian population in the world is continuously increasing. (12, 13) Moreover, Taiwan is ranked among the top three countries in the world with the highest proportion of vegetarians. (12, 14) Diet could play a role in the pathogenesis of VVs. Nevertheless, the impact of dietary patterns on VVs has not been comprehensively investigated and lacks definitive conclusions. This research aimed to investigate whether following a vegetarian diet had an impact on the likelihood of developing varicose veins among Taiwanese men and women.

## Materials and methods

### Data source and study participants

The present study encompassed a sample size of 9905 adults whose data were obtained from the Taiwan Biobank, a population-based repository that recruits volunteers from the Taiwanese population aged 30 to 70 years and excludes individuals with a personal history of cancer. Recruitment for the Taiwan Biobank project began in 2008. The data used in the current study were collected between 2008 and 2020. Volunteers join the Taiwan Biobank data collection process after fulfilling eligibility criteria and signing informed consent forms. Data on personal information, lifestyle choices, anthropometric measurements, medical history, and biochemical markers are gathered through questionnaires and physical and biochemical examinations. The Institutional Review Board of Chung Shan Medical University Hospital approved the current study (CS2-22035).

### Definition of varicose veins and covariates

Information on varicose veins, vegetarian diet, and covariates including, age, cigarette smoking, alcohol drinking, exercise, educational level, and job type was obtained using questionnaires. Adults who reported the presence of mild, moderate, severe, or more severe distorted blood vessels on their limbs within the past month were considered to have varicose veins. Vegetarians were defined as individuals who maintained a vegetarian lifestyle all day long for over 6 months. Covariates such as smoking, drinking, and exercise were categorized into two groups (yes/no). Body mass index (BMI) was grouped into four categories: normal weight (18.5 ≤ BMI < 24), underweight (BMI < 18.5), overweight (24 ≤ BMI < 27), and obesity (BMI ≥ 27 kg/m^2^). Soldiers, teachers, pharmacists, nurses, etc. were considered to be in jobs that required prolonged standing.

### Data analyses

The SAS version 9.4 (SAS Institute, Cary, NC, USA) was used for data analysis. Differences in categorical and continuous variables between men and women were determined with the Chi-square and Student’s t-tests, respectively. Logistic regression was used to determine the association of varicose veins with a vegetarian diet, sex, and other variables. The threshold for statistical significance (α-value) was set at 0.05.

## Results

Differences in demographic characteristics between men and women are described in Table 1. There were 4,142 men and 5,763 women. Study variables differed significantly between men and women (*p* < 0.05). About 12% of men and 35% of women had VVs. Study participants were primarily non-vegetarians (i.e., 91.84% men and 88.24% women).

**Table 1:** Demographic characteristics of participants stratified by sex.

Variables	Men	Women	*p*-value
	(N = 4,142)	(N = 5,763)	
Varicose veins n, %			<0.0001
No	3,641 (87.90)	3,753 (65.12)	
Yes	501 (12.10)	2010 (34.88)	
Vegetarian n, %			<0.0001
No	3,804 (91.84)	5,085 (88.24)	
Yes	338 (8.16)	678 (11.76)	
Age (years)	49.436 ± 0.169	49.561 ± 0.126	0.5512
Smoking n, %			<0.0001
No	2,413 (58.26)	5,530 (95.96)	
Yes	1729 (41.74)	233 (4.04)	
Drinking n, %			<0.0001
No	3,413 (82.40)	5,637 (97.81)	
Yes	729 (17.60)	126 (2.19)	
Exercise n, %			0.0043
No	2,321 (56.04)	3,395 (58.91)	
Yes	1821 (43.96)	2,368 (41.09)	
BMI (kg/m^2^) n, %			<0.0001
Normal weight	1,506 (36.36)	3,377 (58.60)	
Underweight	47 (1.13)	174 (3.02)	
Overweight	1,502 (36.26)	1,353 (23.48)	
Obese	1,087 (26.24)	859 (14.91)	
Education level n, %			<0.0001
Elementary and below	120 (2.90)	353 (6.13)	
Junior and Senior High School	1,364 (32.93)	2,792 (48.45)	
University and above	2,658 (64.17)	2,618 (45.43)	
Job type n, %			0.2906
Non-prolonged standing	1,573 (37.98)	2,249 (39.02)	
Prolonged standing	2,569 (62.02)	3,514 (60.98)	
Systolic blood pressure (mmHg) n, %	121.200 ± 0.249	112.800 ± 0.228	<0.0001
Diastolic blood pressure (mmHg) n, %	76.398 ± 0.166	68.685 ± 0.137	<0.0001
Hormone use	-		-
No	-	4,883 (84.73)	
Yes	-	880 (15.27)	
Parity	-	3.354 ± 0.019	-

BMI: body mass index, n: sample size, BMI categories: normal (18.5 ≤ BMI < 24), underweight (BMI < 18.5), overweight (24 ≤ BMI < 27), and obesity (BMI ≥ 27 kg/m^2^).

Table 2 shows the association of sex and vegetarian diets with varicose veins. Women had a higher risk of varicose veins than men (OR = 3.414, 95% CI = 2.995–3.891). There was no significant relationship between vegetarian diets and varicose veins (OR = 0.966, 95% CI = 0.829–1.124). Nonetheless, its interaction with sex was significant (*p* = 0.0034). Exercise had a protective association with varicose veins (OR = 0.902, 95% CI = 0.814–0.999). Prolonged compared to non-prolonged standing jobs were linked to a higher risk of varicose veins (OR = 1.223, 95% CI = 1.107–1.350).

**Table 2:** Logistic regression analysis showing the association of sex and vegetarian diet with varicose veins.

Variables	OR	95% CI	*P*-value
Vegetarian (ref: No)			
Yes	0.966	0.829–1.124	0.6516
Gender (ref: Men)			
Women	3.414	2.995–3.891	<0.0001
Age (years)			
Smoking (ref: No)	1.006	1.000–1.012	0.0472
Yes	0.787	0.667–0.928	0.0044
Drinking (ref: No)			
Yes	0.966	0.777–1.200	0.7537
Exercise (ref: No)			
Yes	0.902	0.814–0.999	0.0485
BMI (ref: Normal)			
Underweight	0.687	0.499–0.947	0.0216
Overweight	1.013	0.904–1.136	0.8227
Obese	0.931	0.810–1.070	0.3133
Education level (ref: Elementary and below)			
Junior and Senior High School	1.346	1.067–1.699	0.0123
University and above	1.507	1.186–1.913	0.0008
Job type (ref: Non-prolonged standing)			
Prolonged standing	1.223	1.107–1.350	<0.0001
Systolic blood pressure (mmHg)	0.996	0.991–1.000	0.0784
Diastolic blood pressure (mmHg)	0.995	0.987–1.002	0.1412
Interaction: Sex*vegetarian diet	*P*-value = 0.0034		

OR: odds ratio, CI: confidence interval, BMI: body mass index.

Table 3 displays the association between sex and varicose veins in vegetarians and non-vegetarians. Women had a higher risk of varicose veins than men both in the vegetarian (OR = 1.877, 95% CI = 1.270–2.774) and non-vegetarian (OR = 3.674, 95% CI = 3.197–4.223) groups, respectively. According to Table 4, the results of the analyses (for men and men) indicate that vegetarian diets were significantly linked to varicose veins only in men (OR = 1.453 and 95% CI = 1.069 to 1.976).

**Table 3:** Logistic regression analysis showing the association between sex and varicose veins stratified by vegetarian diets.

Variables	Non-vegetarian			Vegetarian		
	OR	95% CI	*P*-value	OR	95% CI	*P*-value
Gender (ref: Men)						
Women	3.674	3.197–4.223	<0.0001	1.877	1.270–2.774	0.0016
Age (years)	1.007	1.001–1.013	0.0308	1.002	0.984–1.020	0.8467
Smoking (ref: No)						
Yes	0.837	0.704–0.995	0.0434	0.441	0.243–0.799	0.0070
Drinking (ref: No)						
Yes	0.948	0.756–1.189	0.6443	1.254	0.578–2.722	0.5670
Exercise (ref: No)						
Yes	0.898	0.805–1.001	0.0519	0.939	0.690–1.278	0.6881
BMI (ref: Normal)						
Underweight	0.694	0.491–0.982	0.0392	0.677	0.292–1.570	0.3632
Overweight	0.988	0.875–1.115	0.8463	1.308	0.917–1.866	0.1387
Obese	0.906	0.782–1.050	0.1881	1.149	0.751–1.758	0.5215
Education level (ref: Elementary and below)						
Junior and Senior High School	1.350	1.053–1.729	0.0178	1.243	0.626–2.469	0.5341
University and above	1.456	1.129–1.877	0.0038	1.961	0.967–3.977	0.0621
Job type (ref: Non-prolonged standing)						
Prolonged standing	1.229	1.106–1.364	0.0001	1.211	0.886–1.657	0.2300
Systolic blood pressure (mmHg)	0.994	0.989–0.999	0.0174	1.008	0.994–1.022	0.2585
Diastolic blood pressure (mmHg)	0.999	0.991–1.007	0.7936	0.963	0.943–0.984	0.0006

OR: odds ratio, CI: confidence interval, BMI: body mass index.

**Table 4:** Logistic regression analysis showing the association between vegetarian diet and varicose veins stratified by sex.

Variables	Men			Women		
	OR	95% CI	*P*-value	OR	95% CI	*P*-value
Vegetarian (ref: No)						
Yes	1.453	1.069–1.976	0.0170	0.861	0.725–1.023	0.0891
Age (years)	1.006	0.996–1.016	0.2615	1.006	0.999–1.013	0.1055
Smoking (ref: No)						
Yes	0.815	0.666–0.998	0.0479	0.739	0.549–0.995	0.0465
Drinking (ref: No)						
Yes	1.023	0.787–1.331	0.8624	0.876	0.592–1.296	0.5069
Exercise (ref: No)						
Yes	0.907	0.740–1.112	0.3472	0.897	0.797–1.011	0.0751
BMI (ref: Normal)						
Underweight	0.901	0.376–2.163	0.8163	0.668	0.474–0.940	0.0206
Overweight	0.970	0.781–1.207	0.7874	1.026	0.896–1.175	0.7096
Obese	0.820	0.634–1.059	0.1287	0.985	0.834–1.165	0.8633
Education level (ref: Elementary and below)						
Junior and Senior High School	0.902	0.513–1.587	0.7214	1.437	1.115–1.853	0.0051
University and above	1.103	0.629–1.933	0.7317	1.578	1.214–2.052	0.0007
Job type (ref: Non-prolonged standing)						
Prolonged standing	1.317	1.074–1.615	0.0081	1.202	1.073–1.348	0.0016
Systolic blood pressure (mmHg)	1.001	0.992–1.011	0.7726	0.993	0.988–0.999	0.0193
Diastolic blood pressure (mmHg)	0.986	0.973–0.999	0.0358	0.999	0.990–1.007	0.7483

OR: odds ratio, CI: confidence interval, BMI: body mass index.

Table 5 shows the risk of varicose veins based on a combined model. With non-vegetarian men as the reference group, vegetarian men had a significantly higher risk of varicose veins (OR = 1.457, 95% CI = 1.072–1.979). Regardless of their vegetarian status, women were at higher risk of varicose veins than men. The OR (95% CI) for the disease was 3.599 (3.140–4.124) among non-vegetarian women and 3.101 (2.528–3.803) among vegetarian women. Supplementary Table 1 presents an overview of the influence of various risk factors on the development of varicose veins, comparing men with and without a vegetarian diet, as well as women. The only risk factor associated with varicose veins among non-vegetarian men was prolonged standing, whereas obesity seemed to be protective. Among men following a vegetarian diet, varicose veins were significantly associated only with smoking, albeit with a protective effect.

**Table 5:** The odds of varicose veins in men and women based on dietary pattern.

Variables	OR	95% CI	*P*-value
Men, no vegetarian diet	1		
Men, vegetarian diet	1.457	1.072–1.979	0.0161
Women, no vegetarian diet	3.599	3.140–4.124	<0.0001
Women, vegetarian diet	3.101	2.528–3.803	<0.0001

OR: odds ratio, CI: confidence interval, BMI: body mass index.

Adjusted for age, smoking, drinking, exercise, weight, educational level, job type, and systolic and diastolic blood pressure.

## Discussion

In this study, it was observed that women in both the vegetarian and non-vegetarian groups exhibited a significantly higher risk of developing VVs compared to men, even after accounting for other variables. Hence, female sex may be considered a notable risk factor for varicose veins, irrespective of vegetarian dietary status. This increased risk in women could potentially be attributed to sex hormones and pregnancy. (15, 16) In studies involving oophorectomized rats, sex hormone depletion affected venous contractility and decreased vein diameter, whereas hormone replacement therapy improved both factors. (17, 18) Another animal study involving female rats revealed that estrogen-mediated pathways could promote venous dilation and lead to more distensible veins. (19) According to Garcia-Honduvilla et al. (2018), varicose veins in women are associated with increased estrogen and progesterone receptors in all tunica layers. (20) The incidence of varicose veins is not so high in men compared to women due to relatively low concentrations of estradiol. (21)

Research has shown that a vegetarian diet can have significant positive effects on cardiovascular and metabolic health, including weight and glycemic control, blood pressure, lipid reduction, and the reversal of atherosclerosis. (22) However, our study revealed that vegetarian men are 1.45 times more likely to develop varicose veins compared to men who consume an omnivorous diet. Interestingly, there is currently no scientific literature supporting the protective effect of a vegetarian diet on venous circulation.”

Concentrations of serum ferritin (a marker for iron deficiency) were significantly lower in male vegetarians than in male omnivores. (23) Individuals who follow a vegetarian diet may be at an increased risk of iron deficiency, potentially resulting in damage to the gastric mucosa and subsequent development of atrophic gastritis, which can negatively impact the absorption of cobalamin through the intrinsic factor. (24) Gilsing et al. (2010) claimed that vegetarians and vegans have much lower concentrations of serum vitamin B12 in comparison to omnivores. (25) In other studies, plasma vitamin B12 was inversely correlated with plasma homocysteine among vegetarians. (26, 27)

There is limited research on the effects of vitamins and minerals on varicose veins. In a previous study, higher levels of vitamin B12 and homocysteine were found in patients with varicose veins compared to controls, although the difference was not significant (28). A Mendelian study (29), however, found that genetically predicted Vit B12 was associated with varicose veins. Homocysteine, a sulfur-containing amino acid formed during methionine metabolism (30) has been implicated in the induction of endothelial dysfunction, inflammation, and vascular wall leakage, and thus, contributes to CVD complications. (31) Nevertheless, further research is necessary to clarify its role in the etiology of varicose veins.

In our study, individuals with prolonged standing jobs had a higher risk of developing varicose veins. According to a study, standing still for more than 30 min may cause venous hypertension accompanied by an inflammatory response that results in endothelial injury and monocyte adhesion in the venous walls. (32)

The diameter of the great saphenous vein (GSV) is a predictor of varicosity and a suggested criterion in the pre-interventional assessment of varicose disorders. (33, 34) However, Gibson et al. (2020) found the GSV diameter to be a poor surrogate for assessing the impact of varicose veins on patients’ quality of life. (35) Furthermore, there is a difference in the diameter and reflux of the GSV between Asians and other populations. For instance, in a study involving Asians and Caucasians with varicose veins, Asians, compared to their Caucasian counterparts had significantly smaller GSV diameters and more venous reflux, extending to the ankle level in most patients. (36) Even though Asians were observed with smaller vein diameters, VVs was more advanced, (36) indicating that early stages of the disease in Asians may be overlooked due to the relatively small size of their veins. This suggests that factors other than vein diameter may contribute to the severity of venous disease in Asians. Additionally, Asians are often diagnosed at later stages due to delayed appearance of hyperpigmentation changes in the medial aspect of their ankle region.

Our findings suggest that women and men who follow a vegetarian diet are at a higher risk of developing varicose veins. As a result, healthcare practitioners should take into consideration the gender and dietary habits of individuals when implementing early and targeted interventions for the prevention and management of this venous disease.

Despite our study having a substantial sample size in examining the link between varicose veins and vegetarian diets, several limitations need to be acknowledged. Firstly, the study was confined to Han Chinese individuals, and thus, the findings may not apply to other ethnic groups. Secondly, our database lacked information on vitamin B12 and homocysteine levels, which may have potentially impacted the results. Additionally, data on medication use, history of blood clots, and damaged veins were not available in the database, which may have affected the validity of our findings. Lastly, varicose veins were assessed using self-reported questionnaires in the Taiwan Biobank, which may have introduced bias into the study.

## Conclusion

In summary, Taiwanese women were found to have a higher risk of varicose veins compared to men, regardless of diet. However, in terms of diet, only men who followed a vegetarian diet were at greater risk for developing VVs. This study suggests that sex-related factors may play a more prominent role in the development of varicose veins than diet-related factors. Nonetheless, clinicians should consider both variables as predisposing factors for varicose veins when managing chronic venous diseases.

## Data availability statement

The data analyzed in this study is subject to the following licenses/restrictions:  The data that support the findings of this study are available from Taiwan Biobank but restrictions apply to the availability of these data, which were used under license for the current study, and so are not publicly available. Data are however available from the corresponding author upon reasonable request and with permission of Taiwan Biobank. Requests to access these datasets should be directed to Y-PL, Liawyp@csmu.edu.tw.

## Ethics statement

This study was approved by the Institutional Review Board of Chung Shan Medical University Hospital (CS2-202035). Taiwan Biobank participants had provided written informed consent during enrollment.

## Author contributions

C-KT, ON, DT, W-YL, and Y-PL conceived and designed the study. W-YL and Y-PL analyzed the data. All authors interpreted the data. C-KT drafted the manuscript. All authors revised, read, and approved the manuscript.

## Funding

This work was partly supported by the Ministry of Science and Technology, MOST (MOST 111-2811-M-040-001; 111-2121-M-040-002) and the Zuoying Branch of Kaohsiung Armed Forces General Hospital, (KAFGH-ZY_A_111013).

## Conflict of interest

The authors declare that the research was conducted in the absence of any commercial or financial relationships that could be construed as a potential conflict of interest.

## Publisher’s note

All claims expressed in this article are solely those of the authors and do not necessarily represent those of their affiliated organizations, or those of the publisher, the editors and the reviewers. Any product that may be evaluated in this article, or claim that may be made by its manufacturer, is not guaranteed or endorsed by the publisher.
