# Supplementary material for: The association between vegetarian diet and varicose veins might be more prominent in men than in women
Source: Front Nutr. 2023 Jun 1;10:1046158. doi: 10.3389/fnut.2023.1046158 (PMC10267867; doi:10.3389/fnut.2023.1046158)
Supplement: Supplementary file 1 [file Table_1.docx]

Supplementary Table 1. The association between related factors and varicose veins in men and women with and without vegetarian diets

| Variables | Men | | | | | | Women | | | | | |
| --- | --- | --- | --- | --- | --- | --- | --- | --- | --- | --- | --- | --- |
|  | Non-vegetarian | | | Vegetarian | | | Non-vegetarian | | | Vegetarian | | |
|  | OR | 95 % CI | P-value | OR | 95 % CI | P-value | OR | 95 % CI | P-value | OR | 95 % CI | P-value |
| Age (years) | 1.007 | 0.996-1.017 | 0.2348 | 1.000 | 0.965-1.037 | 0.9813 | 1.005 | 0.997-1.013 | 0.2214 | 0.999 | 0.977-1.021 | 0.9214 |
| Smoking (ref: No) |  |  |  |  |  |  |  |  |  |  |  |  |
| Yes | 0.866 | 0.700-1.071 | 0.1851 | 0.403 | 0.193-0.839 | **0.0151** | 0.761 | 0.557-1.039 | 0.0860 | 0.428 | 0.145-1.262 | 0.1242 |
| Drinking (ref: No) |  |  |  |  |  |  |  |  |  |  |  |  |
| Yes | 1.037 | 0.790-1.361 | 0.7925 | 0.867 | 0.303-2.480 | 0.7905 | 0.785 | 0.518-1.188 | 0.2520 | 2.964 | 0.787-11.164 | 0.1082 |
| Exercise (ref: No) |  |  |  |  |  |  |  |  |  |  |  |  |
| Yes | 0.938 | 0.757-1.162 | 0.5588 | 0.737 | 0.375-1.450 | 0.3774 | 0.884 | 0.778-1.004 | 0.0569 | 1.027 | 0.722-1.461 | 0.8826 |
| BMI (ref: Normal) |  |  |  |  |  |  |  |  |  |  |  |  |
| Underweight | 1.004 | 0.416-2.423 | 0.9937 | <0.001 | <0.001->999.999 | 0.9872 | 0.664 | 0.457-0.965 | **0.0317** | 0.753 | 0.317-1.790 | 0.5208 |
| Overweight | 0.904 | 0.720-1.137 | 0.3888 | 2.095 | 0.989-4.436 | 0.0535 | 1.017 | 0.881-1.174 | 0.8185 | 1.154 | 0.761-1.749 | 0.5005 |
| Obesity | 0.744 | 0.566-0.977 | **0.0334** | 1.816 | 0.792-4.165 | 0.1590 | 0.984 | 0.824-1.175 | 0.8588 | 0.941 | 0.560-1.581 | 0.8169 |
| Education level (ref: Elementary and below) |  |  |  |  |  |  |  |  |  |  |  |  |
| Junior and senior school | 1.043 | 0.563-1.931 | 0.8936 | 0.249 | 0.048-1.291 | 0.0978 | 1.414 | 1.079-1.852 | **0.0119** | 1.649 | 0.765-3.553 | 0.2017 |
| University and above | 1.185 | 0.642-2.186 | 0.5878 | 0.699 | 0.142-3.445 | 0.6597 | 1.518 | 1.146-2.010 | **0.0036** | 2.326 | 1.044-5.185 | 0.0390 |
| Job type (ref: Non-prolonged standing) |  |  |  |  |  |  |  |  |  |  |  |  |
| Prolonged standing | 1.323 | 1.067-1.641 | **0.0109** | 1.041 | 0.531-2.041 | 0.9068 | 1.200 | 1.064-1.355 | **0.0031** | 1.216 | 0.849-1.741 | 0.2858 |
| Systolic blood pressure (mmHg) | 1.000 | 0.991-1.010 | 0.9607 | 1.003 | 0.973-1.034 | 0.8364 | 0.991 | 0.985-0.997 | **0.0043** | 1.009 | 0.993-1.025 | 0.2532 |
| Diastolic blood pressure (mmHg) | 0.989 | 0.975-1.003 | 0.1232 | 0.967 | 0.928-1.007 | 0.1027 | 1.003 | 0.994-1.013 | 0.4706 | 0.961 | 0.937-0.987 | **0.0031** |
| Hormone use (ref: No) |  |  |  |  |  |  |  |  |  |  |  |  |
| Yes | - | - | - | - | - | - | 1.123 | 0.954-1.321 | 0.1623 | 1.413 | 0.881-2.267 | 0.1520 |
| Parity | - | - | - | - | - | - | 1.022 | 0.980-1.065 | 0.3167 | 1.027 | 0.917-1.150 | 0.6478 |

OR: odds ratio, CI: confidence interval, BMI: body mass index
